# Supplementary material for: The interactome of intact mitochondria by cross-linking mass spectrometry provides evidence for coexisting respiratory supercomplexes
Source: Mol Cell Proteomics. 2017 Dec 8;17(2):216–32. doi: 10.1074/mcp.RA117.000470 (PMC5795388; doi:10.1074/mcp.RA117.000470)
Supplement: Supplemental Data [file supp_RA117.000470_133922_0_supp_23978_fzffwf.zip › spectra_annotation/mito_DR_spectra_annotation/81-1-6-1-5-1.pdf]

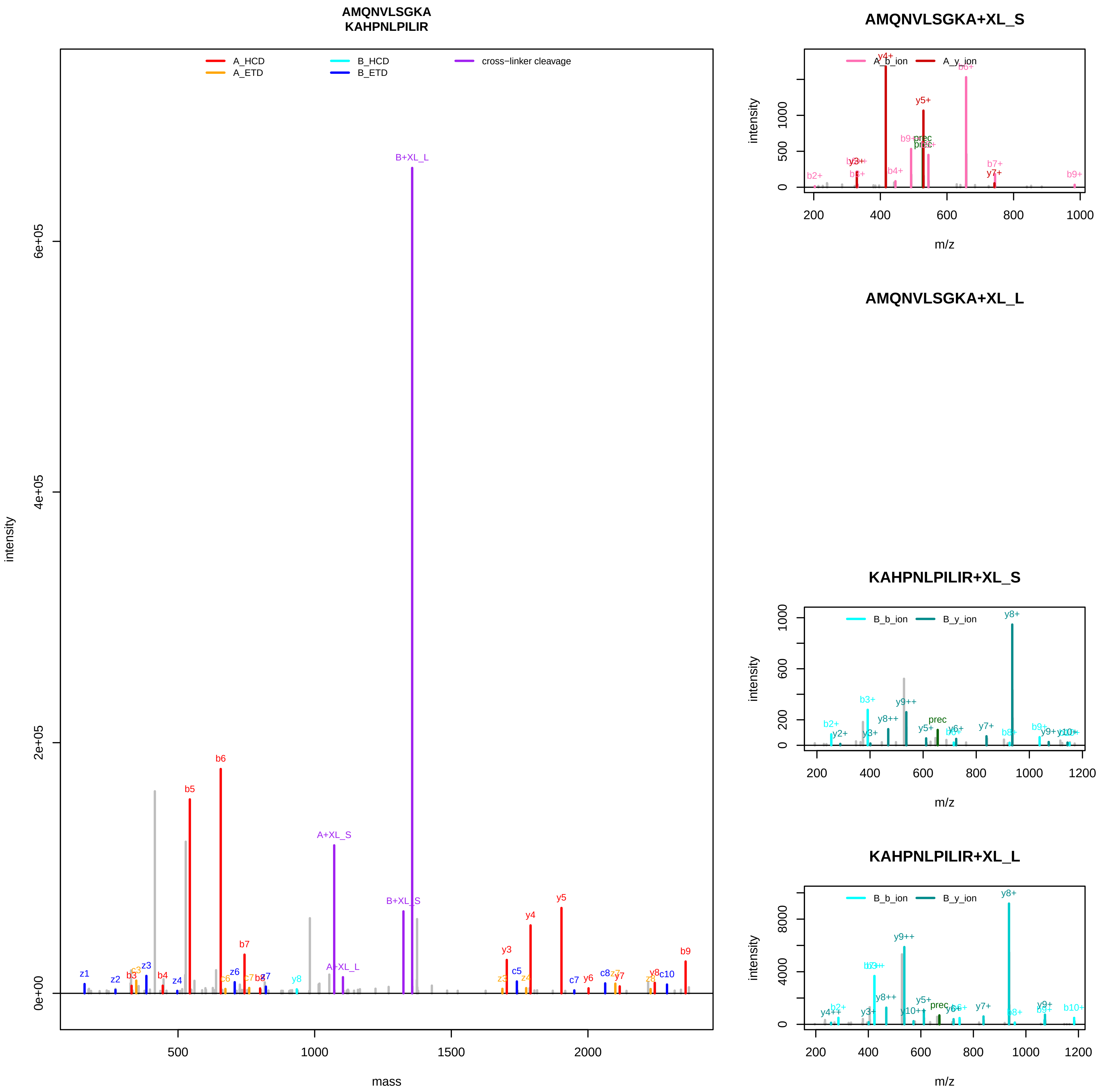
AMQNVLSGKA
KAHPNLPILIR
A_HCD
A_ETD
B_HCD
B_ETD
cross−linker cleavage
B+XL_L
A+XL_S
B+XL_S
A+XL_L
b5
b6
b7
b3
b4
b8
b9
y3
y4
y5
y6
y7
y8
z1
z2
z3
z4
z6
z7
c5
c7
c8
c10
intensity
mass
0e+00
2e+05
4e+05
6e+05
500
1000
1500
2000
AMQNVLSGKA+XL_S
A_b_ion
A_y_ion
y4+
y5+
b6+
b7+
y7+
b9+
b2+
b4+
prec
m/z
AMQNVLSGKA+XL_L
KAHPNLPILIR+XL_S
B_b_ion
B_y_ion
y8+
y9++
y8++
b3+
b2+
y2+
y3+
y5+
y6+
y7+
b8+
b9+
y9+
y10+
KAHPNLPILIR+XL_L
b10+
y4++
y10++
200
400
600
800
1000
1200
